# Cloning, Expression and Characterization of a Novel Thermophilic Polygalacturonase from *Caldicellulosiruptor bescii* DSM 6725

**DOI:** 10.3390/ijms15045717

**Published:** 2014-04-03

**Authors:** Yanyan Chen, Dejun Sun, Yulai Zhou, Liping Liu, Weiwei Han, Baisong Zheng, Zhi Wang, Zuoming Zhang

**Affiliations:** 1 Key Laboratory for Molecular Enzymology & Engineering of the Ministry of Education, School of Life Science, Jilin University, Changchun 130012, China; E-Mails: cyy09@mails.jlu.edu.cn (Y.C.); llp126@126.com (L.L.); weiweihan@jlu.edu.cn (W.H.); zhengbaisong@126.com (B.Z.); wangzhi@jlu.edu.cn (Z.W.); 2 School of Pharmaceutical Sciences, Jilin University, Changchun 130021, China; E-Mails: sundj@jlu.edu.cn (D.S.); zhouyl@jlu.edu.cn (Y.Z.)

**Keywords:** thermophilic polygalacturonase, CbPelA, *exo*-PGase, *Caldicellulosiruptor bescii*

## Abstract

We cloned the gene *ACM61449* from anaerobic, thermophilic *Caldicellulosiruptor bescii*, and expressed it in *Escherichia coli* origami (DE3). After purification through thermal treatment and Ni-NTA agarose column extraction, we characterized the properties of the recombinant protein (CbPelA). The optimal temperature and pH of the protein were 72 °C and 5.2, respectively. CbPelA demonstrated high thermal-stability, with a half-life of 14 h at 70 °C. CbPelA also showed very high activity for polygalacturonic acid (PGA), and released monogalacturonic acid as its sole product. The *V*_max_ and *K*_m_ of CbPelA were 384.6 U·mg^−1^ and 0.31 mg·mL^−1^, respectively. CbPelA was also able to hydrolyze methylated pectin (48% and 10% relative activity on 20%–34% and 85% methylated pectin, respectively). The high thermo-activity and methylated pectin hydrolization activity of CbPelA suggest that it has potential applications in the food and textile industry.

## 1. Introduction

Pectin is a family of acidic complex heteropolysaccharides and is one of the major components of the plant cell wall and middle lamella [1]. There are three known pectic structural elements: homogalacturonans, substituted galacturonans, and rhamnogalacturonan-I [2,3].

The pectin polymer can be degraded by a set of pectinolytic enzymes. Pectinases are classified into one of three major groupings, pectate lyase, polygalacturonase, or pectin methylesterase [1,4,5]. Among these, polygalacturonases (PGases) catalyze the hydrolysis of the homo-galacturonan skeleton (including *endo*-polygalacturonases (*endo*-PGases) and *exo*-polygalacturonases (*exo*-PGases)) [6]. *Endo*-PGases randomly hydrolyze pectin while *exo*-PGases specifically hydrolyze the gylcosidic bonds sequentially from the non-reducing end of the pectin [7]. A number of *exo*-PGases in microorganisms and higher plants have been reported in recent years [7–14]. Depending on the products of reaction, *exo*-PGases are classified into two families: E.C. 3.2.1.67 (*exo*-polygalacturonase, EPG) and E.C. 3.2.1.82 (*exo*-poly-α-galacturonosidase, EPGD) [6]. EPG (mainly produced by fungi or plants) can release monogalacturonic acid from its nonreducing end, while EPGD (mainly produced by bacteria) releases galacturonic acid dimers as its major end product [1,6].

Pectinolytic enzymes play an important role in the fruit juice, paper, and textile industries, as well as for the extraction of oils [15,16]. It is well known that thermophilic enzymes have certain advantages over mesophilic enzymes in that they have a greater ease of purification, higher thermo-stability, and greater chemical resistance, all of which may result in lower costs in industry applications [17]. A number of thermophilic organisms have been discovered in recent years, including a handful of thermophilic pectinases from thermophiles [18–26]. Polygalacturonase has been isolated from *Thermotoga maritima*, *Bacillus* sp. MG-cp-2 [23], and *Sporotrichum thermophile* [22], and pectate lyase has been isolated from *T. maritime* [24], *Thermoanaerobacter italicus* [25], and *Bacillus* sp. TS 47 [26]. However, only two thermophilic EPGs have been isolated from thermophilies [27–29]. One of the thermo-active EPGs that was identified was PelB cloned from *T. maritime.* Its optimal active and melting temperatures are 80 and 105 °C, respectively [18,27]. Another thermophilic pectinase, PecJKR01, was isolated from a soil metagenome sample. PecJKR01 demonstrated activity over a broad range of temperature and pH [29].

Here, we successfully cloned *ACM61449* from *Caldicellulosiruptor bescii* DSM 6725, and expressed it in *Escherichia coli*. We then characterized the resulting recombinant protein, CbPelA, and identified it as a novel thermophilic EPG.

## 2. Results and Discussion

### 2.1. Sequence Alignment and Phylogenetic Analysis of ACM61449 and ACM60667

**Figure 1.**
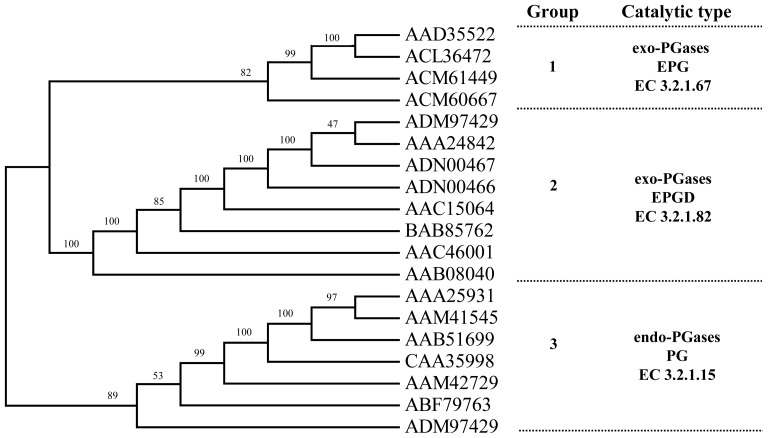
Phylogenetic tree representing a hypothesis of evolutionary relationships for bacterial polygalacturonases (Labels at tree terminals indicate GenBank accession numbers). The confidence scores (percent) of a bootstrap test of 1000 replicates are indicated for major branching nodes.

The genome of *C. bescii* was sequenced previously [30] and ACM61449 and ACM60667 were predicted to be polygalacturonases. Both sequences were subsequently classified into the glycoside hydrolase family 28 (http://www.cazy.org) [31]. We examined the phylogenetic relationships of *ACM61449* and *ACM60667* with other bacterial polygalacturonases (PGases), which sequences that were taken from Genbank. Sequences were aligned using the program Clustal X 1.83 [32]. We then conducted a phylogenetic analysis in Clustal X using the neighbor-joining (NJ) method with 1000 bootstrap replications (Figure 1). PGases from bacteria sorted into three major clades (Figure 1). The three groups are characterized by having different catalytic types (1st group: *exo*-PGases (EPG; EC 3.2.1.67); 2nd group: *exo*-PGases (EPGD; EC 3.2.1.82); 3rd: *endo*-PGases (PG; EC 3.2.1.15)). Our results suggest that *ACM61449* and *ACM60667* belong to the 1st group, and may exhibit EPG activity, similar to what has been reported for AAD35522 (PelB) [18] or ACL36472 (PecJKR01) [29].

**Figure 2.**
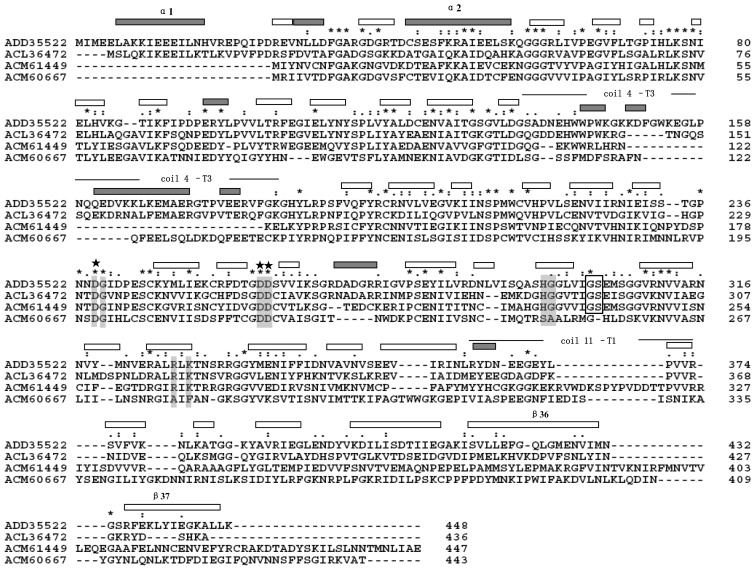
Multiple sequence alignment of bacterial EPGs. The alignment was performed in Clustal X 1.83 with default parameters. Active sites are marked with asterisks. Substrate-binding sites are indicated by a grey background. The secondary structure elements of AAD35522 (PelB) are indicated above the alignment (open square, α-helix; solid square, β-sheet). G230–S231 *cis*-peptide (ACM61499 reference) is indicated by an open square. The strongly conserved positions are marked above the alignment with “*”, “:” and “.”; The residue number of four sequences is indicated on the right, respectively.

The protein sequences of the four members in Group 1 were analyzed through BLAST searches (http://blast.ncbi.nlm.nih.gov/Blast.cgi). We found that ACM61449 shares 37% and 46% sequence identity with ADD35522 and ACL36472, while ACM60667 shares 30% and 34% sequence identity with ADD35522 and ACL36472. A clustal X alignment of the four members showed that three catalytic sites (D181, D202, and D203, ACM61499 reference) were all highly conserved in these four members (Figure 2). Five additional substrate-binding sites (N179, H232, G233, R264, and K266, ACM61499 reference) were conserved in ACM61449 but not in ACM60667, where the residues His, Gly, Arg, and Lys were replaced by Ser, Ala, Ala, and Pro, respectively. Furthermore, the G238-S239 *cis*-peptide motif, which is believed to play an important role in the anchoring of the oriented galacturonate unit to the catalytic sites [18], was also conserved in ACM61449 but not in ACM60667. All these results suggested that ACM61449 may have catalytic behavior similar to that of ADD35522.

### 2.2. Cloning, Expression, and Purification of CbPelA

**Figure 3.**
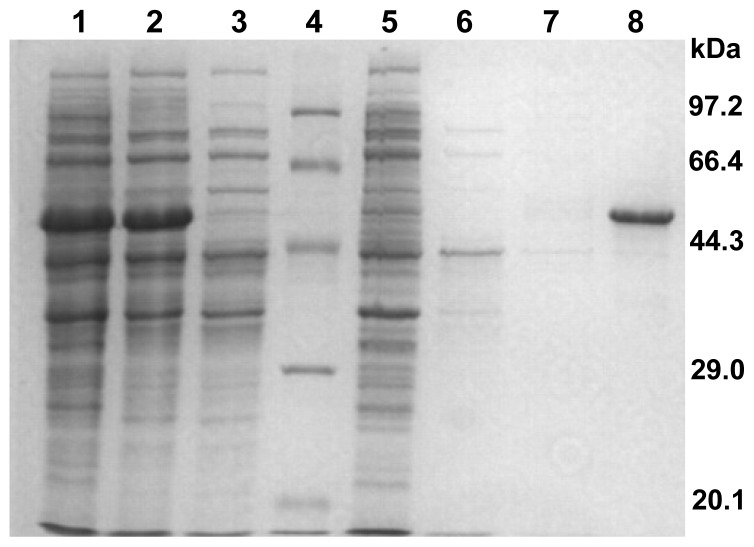
SDS-PAGE gel electrophoresis of the purified CbPelA protein. Lane 1: supernatant of the cell lysate treated by ultrasound; Lane 2: supernatant of the product after thermo-treatment; Lane 3: elution product following filtering through a Ni–NTA agarose column; Lane 4: protein marker; Lanes 5–8: samples eluted with 0, 20, 50, or 100 mM imidazole, respectively. Molecular mass is indicated at the right of the figure.

We extracted the genomic DNA of *C. bescii*and amplified *ACM61449* using PCR. The amplified gene was inserted into a pET20b (+) vector and transformed into *E. coli* origami (DE3). The recombinant protein, CbPelA, was expressed in soluble form following induction with 0.5 mM isopropyl β-d-1-thiogalactoside (IPTG). The cell-free supernatant was incubated at 70 °C for 30 min and then centrifuged. The supernatant was further purified on a Ni-NTA agarose affinity column to obtain the desired protein. Purity was confirmed using examining the protein on a 12% SDS-PAGE gel (Figure 3). The molecular mass of the purified protein was 50 kD, which is congruent with the predicted molecular weight of the mature protein (51 kD).

### 2.3. Enzyme Properties of CbPelA

**Figure 4.**
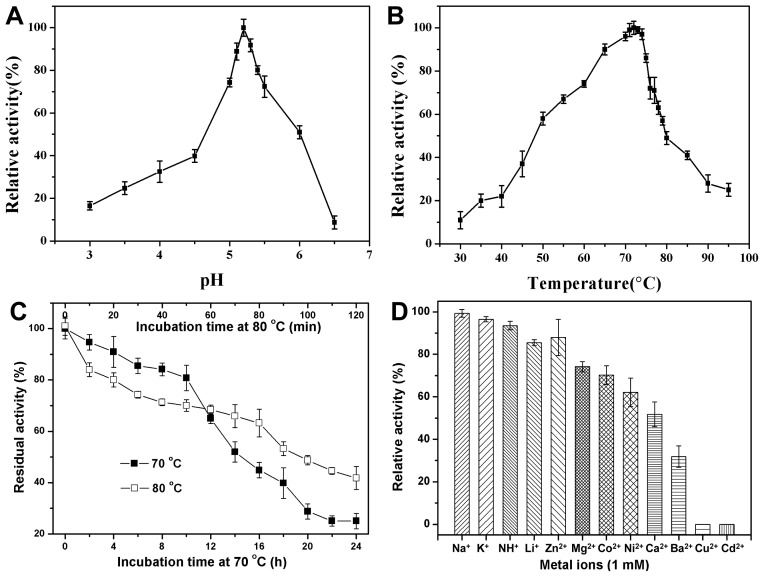
Mean (SD) of various properties of CbPelA. (**A**) Effect of pH; (**B**) Effect of temperature; (**C**) Thermo-stability; (**D**) Effect of metal ions. Mean (SD) was calculated from three independent replicates.

We examined the enzyme properties of CbPelA using polygalacturonic acid (PGA) as a substrate (Table 1; Figure 4).

#### 2.3.1. Effect of pH on CbPelA Activity

The effect of pH on CbPelA activity was determined in 50 mM sodium-acetate buffer with different pHs (3–6.5). We found that the pH-activity curve was narrow (Figure 4A). CbPelA retained approximately 80% of its relative activity between pH 5.0–5.5, which suggests that the activity of CbPelA is also very sensitive to pH. The results also demonstrate that the optimal pH was 5.2. In fact, all reported EPGs exhibit their highest activities in acidic or neutral conditions (Table 1). This may be due to the fact that acidic or neutral conditions are favorable for the enzyme-substrate interactions, and thus increase enzyme activity [35].

#### 2.3.2. Effect of Temperature on CbPelA Activity

We examined the effect of temperature on CbPelA, and found that its activity increased as temperature increased up to 72 °C, with activity then rapidly decreasing (Figure 4B). The CbPelA presented over 90% relative activity in the range of 65 to 75 °C, and the optimal temperature was around 72 °C. To date, only two thermophilic EPGs have been characterized (Table 1). It is known that elevated temperatures are often needed in some industrial processes, for example in the clarification or color extraction steps of fruit juice production, and in textile or plant fiber processing. Thus, the thermophilic EPG we characterized here may be compatible with these needs.

#### 2.3.3. Thermo-Stability of CbPelA

The half-life of CbPelA (1 mg/mL) at 70 and 80 °C was 14 h and 90 min, respectively (Figure 4C). CbPelA showed higher thermal-stability than most reported EPGs (Table 1). The half-life of PecJKR01 at 60 °C is 5 h [29]. The bacterial Pgu B [8] retained about 20% of its activity at 60 °C for less than 10 min. The other EPGs (PG [13] or PGC2 [34]) isolated from eukaryotes showed low thermo-stabilities (Figure 1). The higher thermo-stability of CbPelA that we characterized may be attributed to its low molecular mass (Table 1), which may decrease the solvent-exposed hydrophobic surface area [15]. Furthermore, the *C*-terminal may play a significant role in enzyme thermo-stability [18]. According to our sequence alignment results (Figure 2), CbPelA has an extended *C*-terminus. This may be another reason that CbPelA has a high thermo-stability.

#### 2.3.4. The Effect of Metal Ions on CbPelA Activity

We examined the effect of metal ions on CbPelA activity under optimal temperature and pH conditions. The results indicate that monovalent cations (1 mM) had no obvious effect on enzyme activity (Figure 4D). These results were similar to those reported for other EPGs [8,13]. In contrast, the enzyme activity of CbPelA was highly inhibited by most divalent metal cations we examined. For example, the enzyme activity decreased by 68% and 48% with the addition of Ba^2+^ and Ca^2+^, respectively. CbPelA was completely inhibited when Cu^2+^ and Cd^2+^ were added. However, the effect of divalent metal cations varies greatly among the reported EPGs. For example, Pgu B is completely inhibited by Cu^2+^ [8], while PG is able to retain 50% of its activity [13]. Moreover, Mg^2+^ or Co^2+^ enhances the activity of PG, while Ca^2+^ has no significant effect on both PG and PecJKR01 [13,29]. Therefore, the interaction between the EPGs and divalent metal cations is complex, and further investigation is needed to clarify the mechanism.

### 2.4. Analysis of the Degradation Products of CbPelA

**Figure 5.**
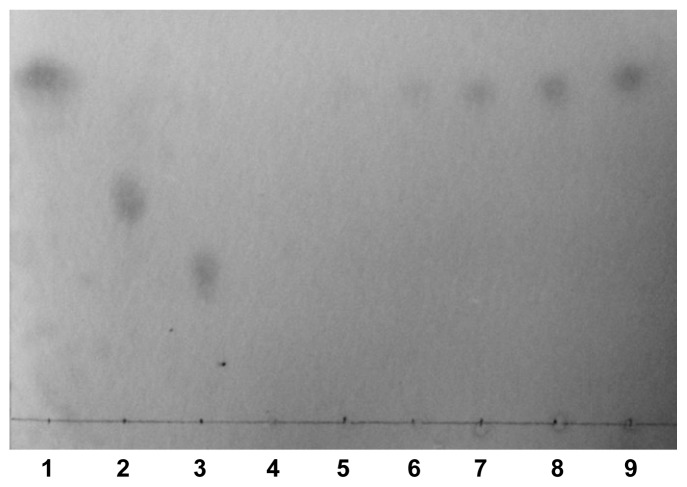
Thin-layer chromatography of the degradation products. Lanes 1–3: Standards of mono-, di-, and tri-galacturonanos, respectively; Lanes 4–9: The products of CbPelA incubated with polygalacturonic acid for different times (0, 1, 2, 4, 8, and 16 h).

The degradation products were analyzed using thin-layer chromatography (TLC) (Figure 5). It was found that galacturonic acid was the only product of degredation, which indicates that CbPelA is an EPG. This result verified another of our analyses (see Section 2.1 above). Until now, only two bacterial EPGs (PelB [27] and Pgu B [8]) had been reported in the literature, while other EPGs were isolated from eukaryotes [6,16] or soil metagenome samples [29]. This, the CbPelA obtained in this study, is only the third EPG to be isolated from bacteria.

### 2.5. Substrate Specificity

CbPelA showed high activity on PGA but had no activity (about 0 U·mg^−1^) on other polysaccharides we tested (such as Avicel, CMC, Xylan, glucan, and soluble starch). These results suggest that CbPelA was specifically active towards the α-1,4-galacturonic acid linkages of galactopolysaccharides. We determined the kinetic characteristics of CbPelA for PGA with a Lineweaver-Burk plot. The *K*_m_ and *V*_max_ were calculated as 0.3 mg·mL^−1^ and 386.8 U·mg^−1^, respectively. The *V*_max_ of CbPelA was lower than that of PelB [27] and PgaX [33], but higher than that of other EPGs (Table 1).

**Figure 6.**
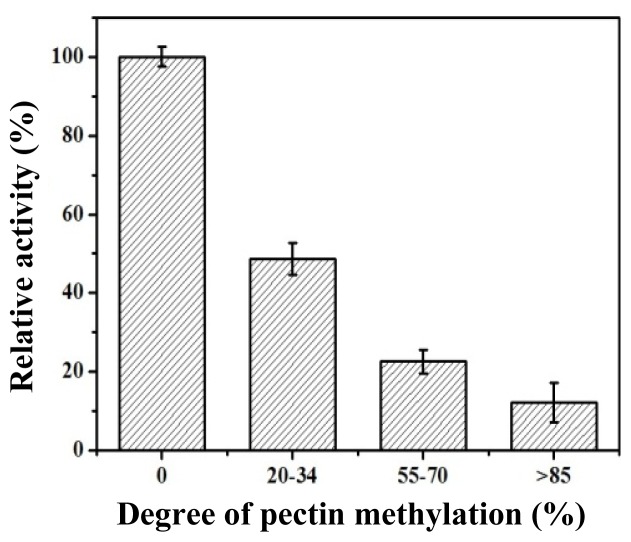
Effect of the degree of pectin methylation on CbPelA activity. Mean (SD) was calculated from three independent replicates.

We also examined the effect of the degree of pectin methylation on CbPelA activity (Figure 6). We found that a higher degree of pectin methylation corresponded to a lower activity of CbPelA. The activity of CbPelA towards PGA was much higher (over five-fold) than that towards high-methylated (>85%) pectin. This result indicates that CbPelA is a polygalacturonase rather than a pectinase, because polygalacturonases typically have unmethylated pectin.

Generally speaking, the bacterial EPGs we examined showed low or no activity towards methylated pectin. For example, Bacterial PelB from *T. maritima* showed low activity on a highly methyl-esterified substrate [27]. PecJKR01 showed no activity on 9% methylated pectin [29]. On the contrary, fungal EPGs commonly exhibit high activity towards methylated pectin. For example, fungal PG [13] isolated from *A. giganteus* showed high activity on 34% methyl-esterified pectin. However, in our study, CbPelA isolated from *Caldicellulosiruptor bescii* bacteria showed high activity on methylated pectin (Figure 4A). The catalytic behavior of this protein was similar to that of EPGs reported from fungi [13,33,34]. The segments of *Caldicellulosiruptor bescii* CbPelA that form the active-site cleft are different than those reported from other bacterial EPGs (Figure 2). This may be one reason why CbPelA’s demonstrates unique methylated pectin hydrolysis. Furthermore, the subtle changes around active-site and/or substrate-binding regions of CbPelA may also contribute to its substrate recognition.

## 3. Methods

### 3.1. Recombinant DNA Techniques

*Caldicellulosiruptor bescii* DSM 6725 was grown anaerobically at 70 °C as described by Yang [36]. The genome of *C. bescii* was isolated following Kataeva *et al.* [30]. The open reading frame of polygalacturonase (ACM61449) was amplified by PCR with PrimeSTAR^®^ HS DNA Polymerase (TAKARA, Dalian, China) and sequence-specific primers (sense 5′-GGAGATATACAT ATGAGAATAATTGTAACTGACT-3′ and antisense 5′-GTGGTGGTGCTCGAGTAGCTACTTTTC TAA-3′; *Nde* I and *Xho* I sites in bold). The following PCR program was used for amplification: 5 min of denaturing at 98 °C, followed by 30 cycles of: denaturing (10 s at 98 °C), annealing (15 s at 58 °C), and polymerization (1.5 min at 72 °C). The program was completed by a final polymerization step (10 min at 72 °C). The PCR product and plasmid pET20b were digested separately with *Nde* I and *Xho* I (TAKARA, Dalian, China) restriction enzymes. Following agarose gel purification, the digested fragments were ligated with T4 DNA ligase. The recombinant plasmid, pCbPelA, was then transformed into *E. coli* origami (DE3) (Novagen, Madison, WI, USA) in LB with 50 μg·mL^−1^ ampicillin.

### 3.2. Expression and Purification of pCbPelA

*E. coli* origami (DE3) harboring pCbPelA was grown to an OD_600_ of 0.6 at 37 °C in LB medium, and was then induced with 0.5 mM isopropyl β-d-1-thiogalactoside (IPTG) at 28 °C for 16 h. Cells were harvested, washed twice, and then resuspended in buffer (20 mM Tris–HCl, pH 8.0). The cells were lysed by ultrasonication and cleared by centrifugation. The supernatant was incubated at 70 °C for 30 min and then centrifuged at 16,000× *g* for 10 min. The supernatant was then loaded on a Ni^2+^-NTA-agarose column (Qiagen, Hilden, Germany) that had been equilibrated with 20 mM Tris–HCl buffer (pH 8.0) and eluted by the same buffer containing a linear gradient (20–200 mM) of imidazole. The eluted enzyme was dialyzed gradually against 20 mM Tris–HCl (pH 8.0). SDS-PAGE gel electrophoresis was performed with a 12% acrylamide gel stained with Coomassie brilliant blue R-250 (Sigma Aldrich, St. Louis, MO, USA) [37]. Molecular masses were estimated with a Protein MW Marker (TAKARA, Dalian, China). The protein concentration was defined using a Bradford assay, with bovine serum albumin as the standard [38].

### 3.3. Enzyme Activity Assay

For assaying enzyme activity, CbPelA solution was added to PGA (0.2%, *w*/*v*) in sodium acetate buffer, 50 mM, pH 5.2 and incubated at 70 °C for 5 min. The reaction was stopped by adding 3,5-dinitrosalicylic acid reagent (DNS). Following centrifugation for removing the undissolved substrate, reducing sugars were measured using Miller’s method [39], and galacturonic acid was used as the standard. One unit of activity of polygalacturonase was defined as the amount of enzyme that catalyzed the liberation of 1 μmol galacturonic acid per minute under optimal conditions.

### 3.4. Enzyme Characterization of CbPelA

The effect of temperature on CbPelA activity was carried out by incubating the enzyme with PGA (pH 5.2) at different temperatures ranging from 30 to 95 °C for 5 min. For the determination of optimal pH, PGA was first dissolved in 50 mM of sodium-acetate buffer with varying pHs, and CbPelA was then incubated with substrate solution at 70 °C for 5 min. The influence of metal ions on enzyme activity was tested using above methods with metal ions added. The final concentration of metal ions in the reaction mixture was 1 mM. For thermal-stability determination, CbPelA (1 mg/mL) in 50 mM sodium-acetate buffer was incubated at 70 and 80 °C, respectively. A volume of 20 μL of enzyme solution was removed at the indicated time points and cooled on ice. The residual activity was determined as described above.

To determine substrate specificity, polygalacturonic acid, citrus pectin with three different degrees of methylation (20%–30%, 55%–74%, and >85%, Sigma Aldrich, St. Louis, MO, USA), and other polysaccharides were used as substrates. The enzyme activity on indicated substrates was tested.

The kinetic constants *K*_m_ and *V*_max_ of CpPelA for PGA were determined using Lineweaver-Burk double-reciprocal plots, which were generated by plotting the reciprocal of reaction velocity (1/*V*) against the reciprocal of the corresponding substrate concentrations (1/*S*).

### 3.5. Thin Layer Chromatography

We examined degradation products by thin-layer chromatography (TLC). CpPelA was incubated with PGA and stopped by boiling for 5 min. Samples were spotted on silica gel plate and chromatography was performed with 1-butanol:distilled water:acetic acid (5:3:2 (*v*/*v*)) as the solvent [40]. The desiccated plate was then sprayed with 0.5% orcinol (*v*/*v*) dissolved in 5% (*v*/*v*) sulfuric acid in ethanol followed by a 5-min heat treatment at 105 °C. Galacturonic acid (G_1_), digalacturonic acid (G_2_), and trigalacturonic acid (G_3_) (Sigma Aldrich, St. Louis, MO, USA) were used as standards.

## 4. Conclusions

In this paper, the *ACM61449* was cloned from anaerobic thermophilic *Caldicellulosiruptor bescii* and then expressed in *E. coli* origami (DE3). We purified CbPelA, characterized its properties, and identified it as a new thermophilic *exo*-PGase (EPG; EC 3.2.1.67). The recombinant enzyme exhibited high thermo-stability and achieved maximum activity when using polygalacturonic acid (PGA) as substrate. Furthermore, CbPelA showed strong activity on methylated pectin. The catalytic behavior of the protein was quite different from most bacterial *exo*-PGases, but was similar to fungal EPGs. These properties suggest that this novel enzyme is a promising candidate for industrial processes, such as bioenergy and food production.

## Figures and Tables

**Table 1.** Properties of CbPelA and other EPGs.

Organism	Name	Molecular mass (kDa)	pH optimum	Temperature optimum (°C)	*V*_max_ (U/mg)	Reference
*Caldiceelulosiruptor bescii*	PelA	50	5.2	72	384.6	This study
*Thermotoga maritime*	PelB	50	6.4	80	1170	[27]
*Metagenome*	PecJKR01	47.9	7.0	70	-	[29]
*Klebsiella* sp. *Yl*	PguB	72	6.0	40–50	47.0	[8]
*Aspergillus tubingensis*	PgaX	78	4.2	30	602.8	[33]
*Aspergillus giganteus*	PG	69.7	6.0	55–60	255	[13]
*Fusarium oxysporum*	Pgc2	63	5.0	50	95.24	[34]
